# Bactericidal and bacteriostatic antibiotics and the Fenton reaction

**DOI:** 10.1111/1751-7915.12120

**Published:** 2014-03-07

**Authors:** Carlos Molina-Santiago, Juan L Ramos

**Affiliations:** 1 CSIC-EEZGranada, Spain; 2 Abengoa ResearchAvenida de la Energía Solar 1, 41011, Sevilla, Spain

Recent articles in *Science* by Liu and Imlay (2013), and Keren and colleagues (2013) question whether bactericidal antibiotics in *Escherichia coli* act via the production of reactive oxygen species (ROS). This hypothesis contradicts the view, proposed by Kohanski and colleagues (2007), that bactericidal antibiotics kill bacteria through hydroxyl radical-mediated DNA damage. Kohanski and colleagues (2007) came to this conclusion because in *E. coli*, bactericidal compounds, but not bacteriostatic compounds, lead to the quenching of intrinsic hydroxyphenyl fluorescein (HPF) dye fluorescence. Furthermore, the authors assumed that this quenching was due to the production of hydroxyl radicals. This belief in an ROS-mediated bactericidal mechanism has now been challenged (Keren *et al*., 2013; Liu and Imlay, 2013) through recent observations where bactericidal activity was not disrupted in the absence of oxygen, bactericidal antibiotics do not induce hydrogen peroxide formation and no oxidative stress response is provoked. Liu and Imlay (2013) also proposed that the observed HPF quenching by bactericidal antibiotics is due to oxidation of the dye by high-valence iron (FeO^2+^) initially formed by the Fenton reaction rather than the hydroxyl radical decomposition product.

**Figure 1:**
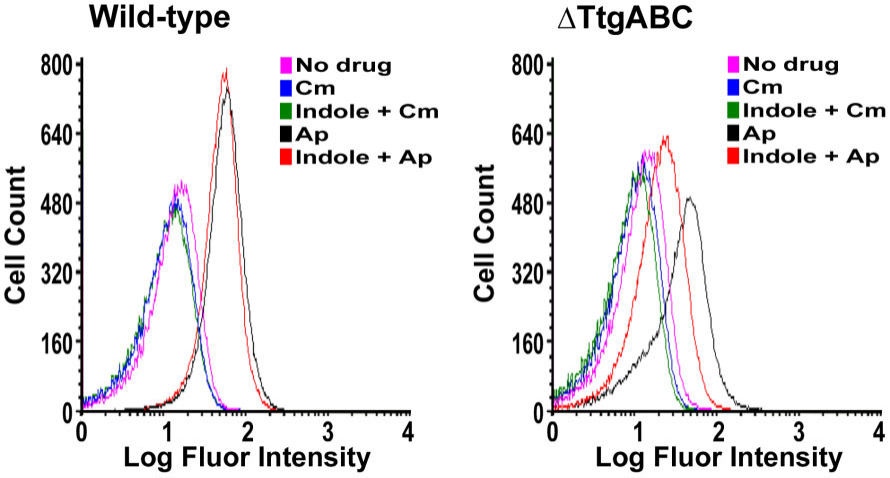
HPF fluorescence quenching in *P. putida* DOT-T1E and DOT-T1E-18 (ΔTtgABC) in the presence and absence of 300 μM indole following exposure to 300 or 150 μg ml^−1^ chloramphenicol (Cm), or 800 or 200 μg ml^−1^ ampicillin (Ap) respectively. Pink, no drug; blue, Luria-Bertani (LB) + chloramphenicol; green, indole + chloramphenicol; black, LB + Ap; red, indole + Ap.

*Pseudomonas putida* is resistant to the bactericidal activity of ampicillin and the bacteriostatic activity of chloramphenicol. This resistance is achieved via TtgABC efflux pump-mediated extrusion of the antibiotics (Godoy *et al*., 2010). Global transcriptional array experiments were carried out and show that similarly, the oxidative stress programme is not initiated in these cells when exposed to the antibiotics (Fernandez *et al*., 2012). HPF dye fluorescence quenching was also observed, but only in the presence of ampicillin and not chloramphenicol (Molina-Santiago *et al*., 2013). In a TtgABC-deficient mutant, the burst of HPF fluorescence quenching was enhanced. When a secondary antibiotic efflux pump, named TtgGHI (Fernandez *et al*., 2012), was induced by indole, a decrease in fluorescence quenching was observed (Molina-Santiago *et al*., 2013) – thus confirming that efflux pump-mediated extrusion of antibiotics influence the Fenton reaction (Fig. 1). Therefore, the hypothesis performed by Liu and Imlay (2013) and Keren and colleagues (2013) where ROS are not exclusively responsible for antimicrobial activity produced by bactericidal compounds can be expanded to other gammaproteobacteria according to our results. Future research is required to determine whether the high-valence FeO^2+^ formed in the presence of bactericidal compounds enhances killing in addition to the direct inhibition of cell wall assembly, protein synthesis or interference with DNA metabolism.
